# Streptococcal Toxic Shock Syndrome Due to Invasive Group A Streptococcal M1UK Strain Infection in a Previously Healthy Child

**DOI:** 10.7759/cureus.77469

**Published:** 2025-01-15

**Authors:** Shin Tsubokura, Norihiko Tsuboi, Tatsuki Ikuse, Goro Koinuma, Hiroki Miyano, Shotaro Matsumoto, Satoshi Nakagawa

**Affiliations:** 1 Critical Care Medicine, National Center for Child Health and Development, Tokyo, JPN; 2 Pediatric Infectious Diseases, National Center for Child Health and Development, Tokyo, JPN; 3 Pulmonology, National Center for Child Health and Development, Tokyo, JPN; 4 Pediatrics, Juntendo University Nerima Hospital, Tokyo, JPN

**Keywords:** bronchitis, group a streptococcus pyogenes, necrotizing pneumonia, pediatric emergency medicine, streptococcal toxic shock syndrome

## Abstract

Invasive group A streptococcal (iGAS) infections are known to be potentially life-threatening. Few detailed pediatric cases of streptococcal toxic shock syndrome (STSS) caused by iGAS with the M1_UK_ strain have been reported. This report describes the case of a child with STSS due to M1_UK_ strain, with detailed documentation of the treatment progress. A 10-year-old female patient without predisposing factors associated with iGAS, initially presented with pneumonia and developed progressive multi-organ failure. A precise diagnosis by the primary hospital’s attending physician and effective critical care in the pediatric intensive care unit (PICU) at a tertiary children’s hospital led to lifesaving and favorable functional outcomes. The clinical course highlights the importance of recognizing the common presentation of iGAS and prompt medical coordination between general hospitals and PICU. Furthermore, public health measures against iGAS infection are just as important as early diagnosis and treatment to prevent deaths in the community after rapid deterioration.

## Introduction

Invasive group A streptococcal (iGAS) infections are known to be potentially life-threatening. Lynskey et al. reported that a new strain, designated M1_UK_, produces more streptococcal pyrogenic exotoxin A than other M1-type strains [1]. In recent years, an increase in the number of patients with iGAS infection has been reported in several countries along with the international spread of the M1_UK_ strain [2], while there have been few reported cases in detailed clinical course and treatment of streptococcal toxic shock syndrome (STSS) due to iGAS with the M1_UK_ strain, especially from Japan. According to surveillance reports in Japan, there has been an increase in reports of STSS since late 2023, similar to trends observed in other countries. This increase encompasses both pediatric and adult cases. Furthermore, a study of the streptococci detected in STSS cases revealed that 51.5% of them were M1_UK_ strains [3]. Matsui et al. reported a single-center pediatric iGAS case series in Japan from 2018 to 2024, which did not include any previously healthy children with STSS caused by the M1_UK_ strain [4]. Despite existing epidemiological reports, detailed clinical documentation of pediatric STSS cases caused by the M1_UK_ strain in Japan remains limited. This report presents a detailed account of the clinical course and treatment of a child with STSS due to the M1_UK_ strain.

## Case presentation

**Figure 1:**
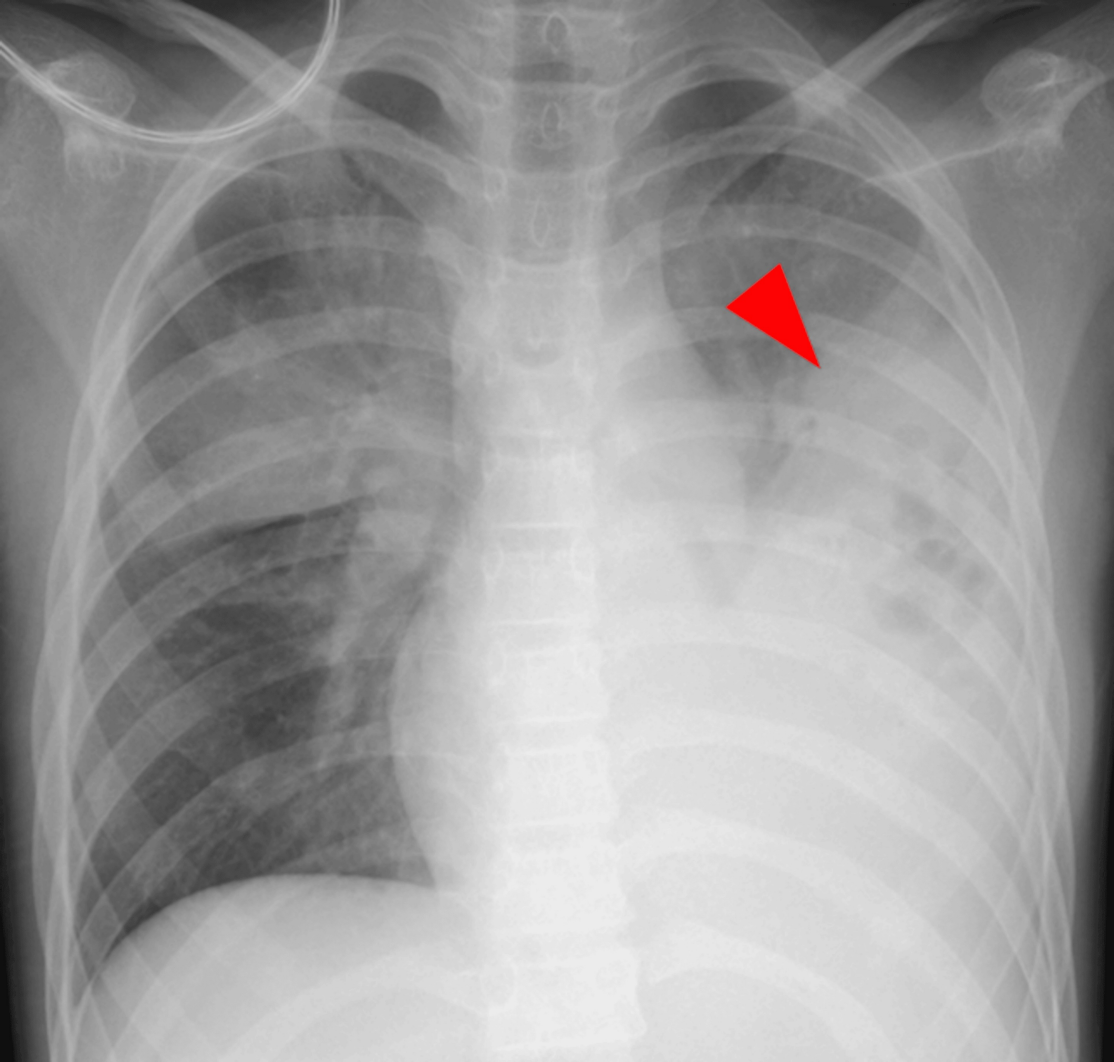
Chest X-ray taken four days before transfer to our pediatric intensive care unit. Chest X-ray revealed lobar pneumonia (arrowhead).

A 10-year-old previously healthy female patient initially presented with a fever four days before transfer to our pediatric intensive care unit (PICU) in June 2024. The patient was hypoxemic and a chest X-ray (Figure 1) revealed lobar pneumonia. The patient’s complete blood count revealed a white blood cell count of 2.6 × 10⁹/L, a hemoglobin level of 13.6 g/dL, and a platelet count of 112 × 10⁹/L. Biochemical analysis showed a markedly elevated C-reactive protein (CRP) level of 335 mg/L. Coagulation studies indicated abnormalities, with a prolonged activated partial thromboplastin time (APTT) of 57.5 seconds, a prothrombin time-international normalized ratio (PT-INR) of 1.7, a fibrinogen level of 876 mg/dL, and a D-dimer level of 31.0 μg/mL. Rapid antigen detection test for group A streptococcus (GAS) was positive from a throat sample. These findings raised suspicion of iGAS infection, so the patient was admitted to the primary hospital and was initially treated with cefotaxime, clindamycin, and intravenous immunoglobulin therapy. The following day, the patient had to be intubated for mechanical ventilation. Blood cultures revealed GAS bacteremia and sputum culture also detected multiple bacteria including GAS. There were no signs of necrotizing fasciitis. STSS was diagnosed. The patient presented with pediatric acute respiratory distress syndrome (PARDS) [5], septic shock, acute kidney injury, and disseminated intravascular coagulation. The patient required sputum aspiration with bronchoscopy, norepinephrine infusion, and fresh-frozen plasma transfusion.

**Figure 2:**
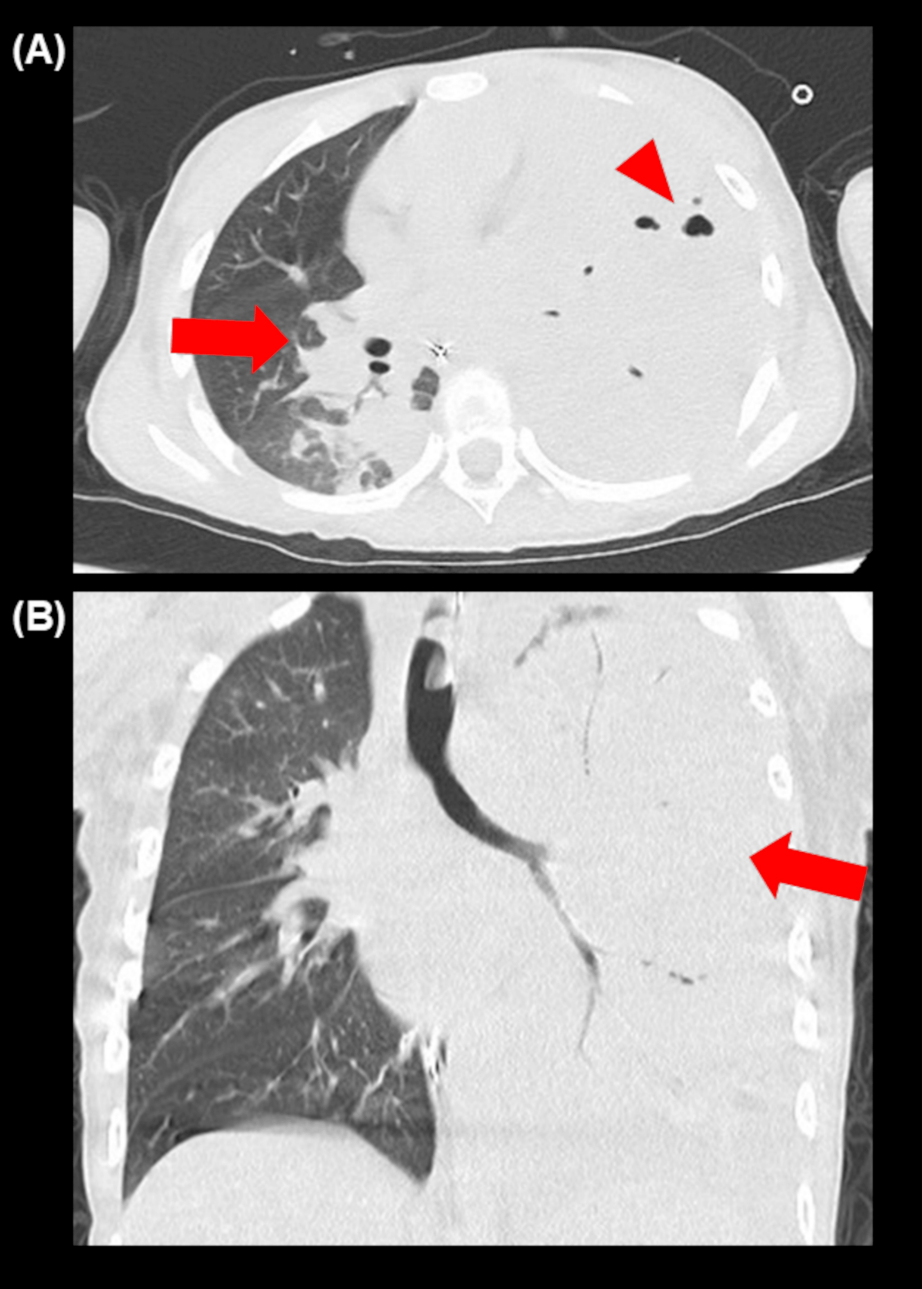
Computed tomography scan performed on the day before transfer to our pediatric intensive care unit. Computed tomography scan showed cavitation in the left lobe (A, arrowhead) and massive consolidation in the right (A, arrow) and left (B, arrow) lobes.

A computed tomography (CT) scan (Figure 2) showed massive consolidation in the right upper and left lower and upper lobes, cavitation destroying the left lower lobe, and left pleural effusion. As the patient’s respiratory failure was so severe that the patient might require extracorporeal membrane oxygenation (ECMO), the patient was transferred to our PICU. Rapid multiplex polymerase chain reaction (PCR) test for respiratory viruses was negative. On admission, the patient required high levels of ventilatory support, though other organs were recovering from failure. The oxygenation index was 9.3, which met the criteria for mild-to-moderate PARDS [5], and lung-protective ventilation was initiated.

**Figure 3:**
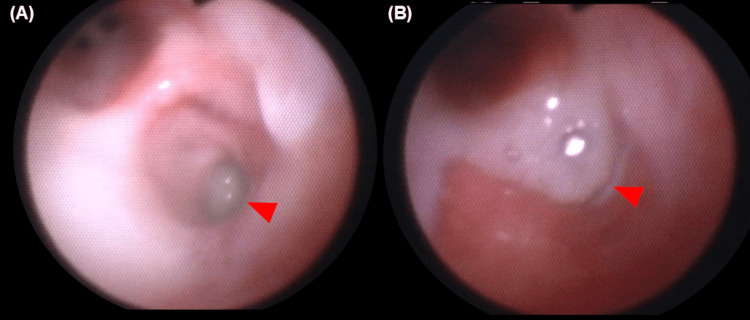
Bronchoscopy images during pediatric intensive care unit stay. Bronchoscopy revealed bronchial obstruction by sputum (A, arrowhead), rising from the lower airways (B, arrowhead).

As a bronchoscopy revealed bronchial obstruction by sputum, daily bronchoscopic sputum suctioning (Figure 3) and chest physical therapy were performed. We used benzylpenicillin potassium instead of cefotaxime because it is known as the first-line treatment regimen of STSS due to iGAS [6]. Clindamycin was continued to be administered for toxin suppression. The next day, a chest tube was inserted to drain the left exudative pleural effusion to prevent progression to pyothorax. These treatments gradually improved the patient’s condition such that ECMO treatment was avoided. On PICU day eight, the patient was extubated, and three days later, the patient was discharged from the PICU.

**Figure 4:**
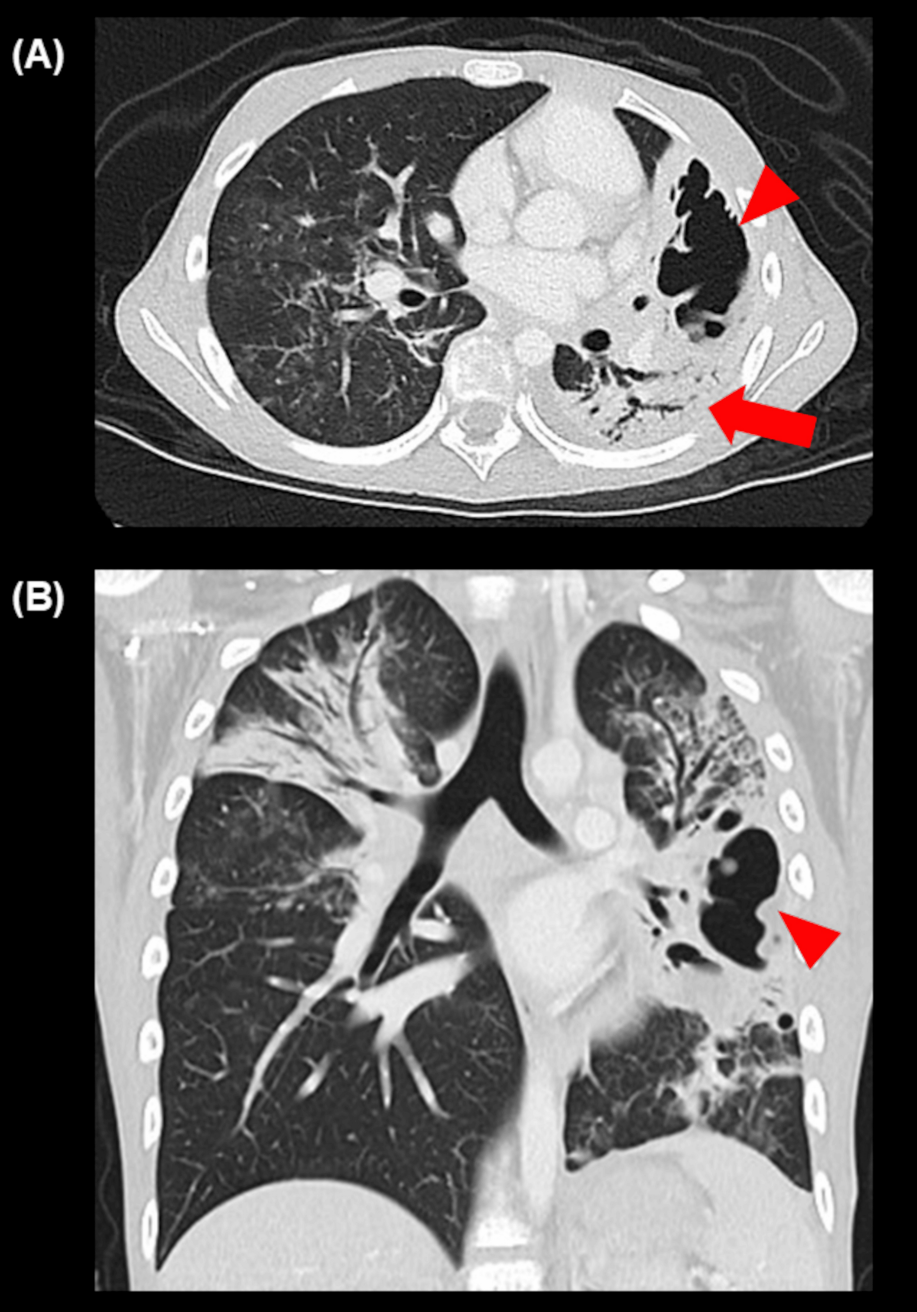
Computed tomography scan one week after discharge from the pediatric intensive care unit. Computed tomography scan showed residual consolidation (A, arrow) and cavitation (A, B, arrowhead).

One week after discharge from the PICU, oxygen therapy was discontinued though a follow-up CT scan (Figure 4) showed residual consolidation and cavitation.

**Figure 5:**
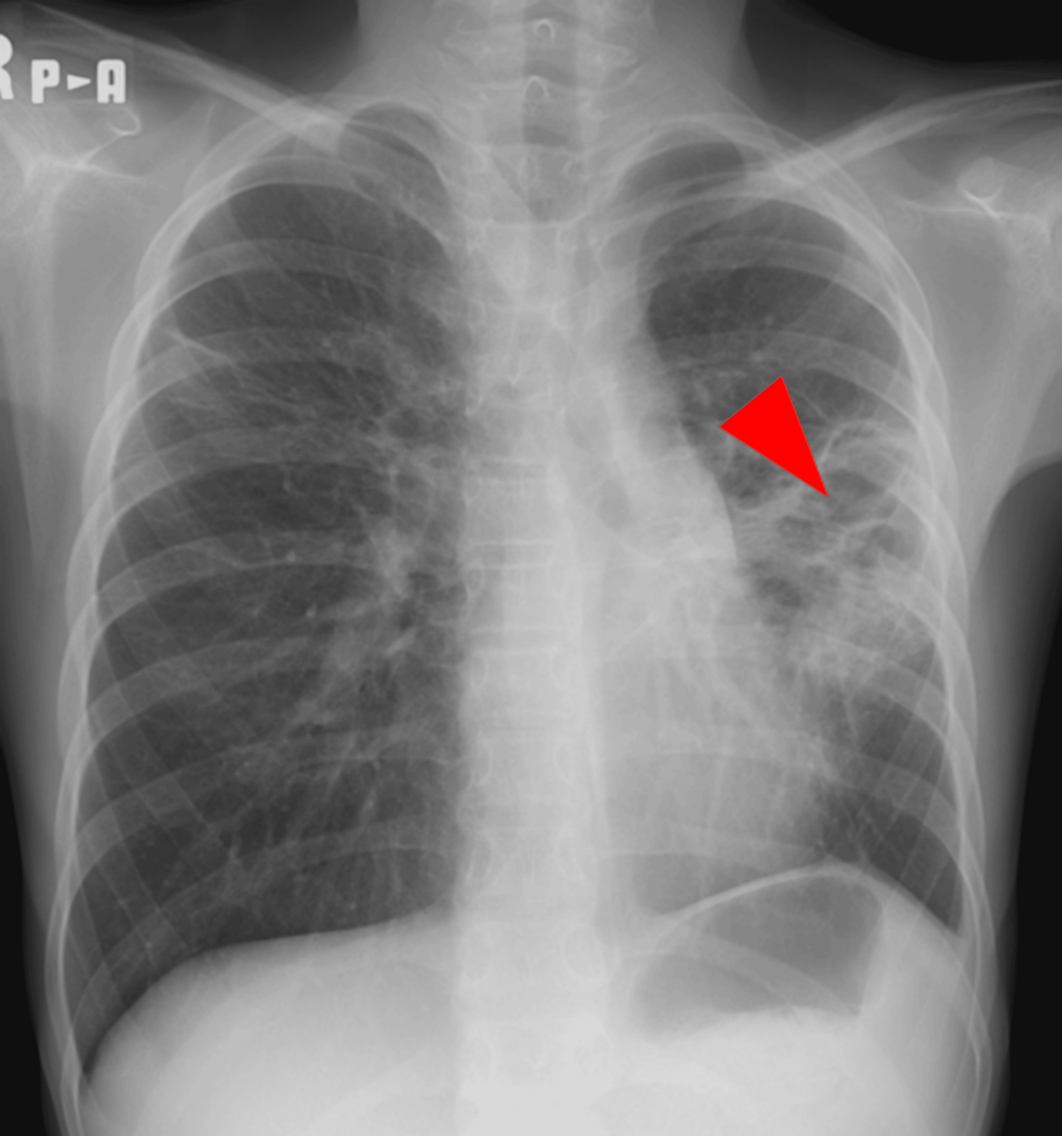
Chest X-ray at hospital discharge. Chest X-ray showed improved consolidation in the left lobe (arrowhead).

The patient continued to undergo comprehensive rehabilitation and was discharged on hospital day 35 without major sequelae. Chest X-ray at hospital discharge(Figure 5) showed improved consolidation in the left lobe. According to the national guideline [7], the allele-specific PCR method [8] was performed in the public laboratory and identified the M1_UK_ strain.

## Discussion

We presented a child with STSS due to iGAS with the M1_UK_ strain. Precise diagnosis by the primary hospital’s attending physician and effective critical care in the PICU led to lifesaving and favorable functional outcomes.

There is a range of clinical presentations related to iGAS, and they are usually non-specific, posing a diagnostic challenge for clinicians [9]. Previous studies have reported that pneumonia was the most frequent clinical condition of pediatric iGAS infection (25-44%) [10, 11], and our patient had pneumonia at the initial presentation. It led to the early diagnosis that the primary hospital’s attending physician recognized lobar pneumonia as a symptom of iGAS infection precisely.

Regarding critical care, it has been reported that 21-39% of children with iGAS infection required admission to intensive care units [10-12], and respiratory distress was significantly associated with severity in multivariate analysis [12]. In the current case, although respiratory failure progressed after the initial treatment, the patient received effective intensive care without delay because of prompt medical coordination between the primary hospital and our PICU. While the case of PARDS with iGAS managed with respiratory ECMO has been reported [13], we selected less invasive treatments. In terms of long-term effects and post-intensive care syndrome in pediatrics [14], Thielemans, et al. reported that 15% of the children with iGAS infection had persistent health problems six months after discharge [10]. Rodríguez et al. also reported 26.8% of the children with STSS survived with sequelae after PICU stay [15]. Our patient was discharged without major sequelae, and the vital capacity (VC) was nearly normal (79% of predicted VC) two weeks after hospital discharge.

Matsui et al. reported that 55% of pediatric iGAS infection patients had underlying diseases [4]. The current patient had no predisposing factors associated with iGAS, such as other viral infections, skin disruption from trauma, immunodeficiency, malignant neoplasm, and an age of less than one year [9]. The clinical course of our case also suggests that community-acquired infection with the M1_UK_ strain leading to life-threatening multi-organ failure can occur in any child. Wrenn et al. reported that the mortality of pediatric iGAS infection in the winter of 2022 was 25%, and 57% of deaths occurred in the community after rapid deterioration, suggesting that public health measures against iGAS infection are required in addition to early diagnosis and treatment [16]. It is important to inform the public about the increase in iGAS among children and that GAS is transmitted through respiratory droplets and direct contact. As stated in several national guidelines [17,18], the effectiveness of droplet and contact precautions such as hand hygiene in the home and community should be announced to raise awareness about infection prevention.

## Conclusions

We reported the previously healthy child with STSS due to iGAS with the M1_UK_ strain referring to detailed treatment progress. Precise diagnosis and effective critical care led to a favorable outcome. The clinical course highlights the importance of recognizing common presentation of iGAS and prompt medical coordination between general hospitals and PICU. The findings could propose appropriate management of pediatric STSS due to iGAS with the M1_UK_ strain in the context of limited detailed case reports. Furthermore, the current case suggested community-acquired infection with the M1_UK_ strain leading to serious multi-organ failure can occur in any child. This report could remind health professionals that public health measures against iGAS infection are just as important as early diagnosis and treatment.
